# The role of the dorsomedial hypothalamus in the cardiogenic sympathetic reflex in the Sprague Dawley rat

**DOI:** 10.3389/fphys.2024.1479892

**Published:** 2024-12-24

**Authors:** Matthew R. Zahner, Kynlee J. Hillard, Michelle C. Chandley

**Affiliations:** ^1^ Health Sciences Department, College of Public Health, East Tennessee State University, Johnson City, TN, United States; ^2^ Biomedical Science Department, Quillen College of Medicine, East Tennessee State University, Johnson City, TN, United States

**Keywords:** cardiogenic sympathetic reflex, renal sympathetic nerve activity, cardiovascular regulation, bradykinin, dorsomedial hypothalamus

## Abstract

Myocardial ischemia causes the production and release of metabolites such as bradykinin, which stimulates cardiac spinal sensory afferents, causing chest pain and an increase in sympathetic activity referred to as the cardiogenic sympathetic afferent reflex. While the brain stem nuclei, such as the nucleus tractus solitarius and rostral ventrolateral medulla, are essential in the cardiogenic sympathetic afferent reflex, the role of other supramedullary nuclei in the cardiogenic sympathetic afferent reflex are not clear. The dorsomedial hypothalamic nucleus (DMH) is involved in cardiovascular sympathetic regulation and plays an important role in the sympathetic response to stressful stimuli. In this study, we determined the role of DMH in the cardiogenic sympathetic afferent reflex. To do this we measured arterial pressure, heart rate, and renal sympathetic nerve activity (RSNA) responses to epicardial bradykinin (10 μg/mL) in anesthetized Sprague Dawley rats before and after bilateral DMH microinjection (50 nL) of either the GABAA agonist muscimol (0.5 nmol) to inhibit or the antagonist bicuculline (40 pmol) to disinhibit activity. Muscimol inhibition elicited a modest, albeit significant, reduction in basal arterial pressure and heart rate and attenuated the arterial pressure and heart rate reflex response to epicardial bradykinin. However, it did not change the magnitude of the reflex. Bicuculline disinhibition of the DMH increased basal arterial pressure, heart rate, and RSNA but did not augment the response to epicardial bradykinin. These results suggest that sympathetic activity derived from the DMH does not play an important role in the cardiogenic sympathetic afferent reflex in Sprague Dawley rats.

## 1 Introduction

The cardiogenic sympathetic afferent reflex is a cardiovascular reflex characterized by a sympathetically mediated increase in blood pressure and heart rate to maintain coronary perfusion. Myocardial ischemia produces several metabolites, including bradykinin (Pan et al., 2000), which activates nerve endings on the epicardial surface of the heart to evoke the cardiogenic sympathetic afferent reflex (Baker et al., 1980; Lombardi et al., 1982; Veelken et al., 1996; Tjen-A-Looi et al., 1998; Zahner and Pan, 2005; Wang et al., 2014; Foreman et al., 2015; Wang et al., 2017; Shanks et al., 2018; Yoshie et al., 2018; Moore, 2024). During myocardial ischemia, the cardiogenic sympathetic afferent reflex may be necessary to maintain vascular flow to coronary vessels. However, the increased sympathetic outflow can also be detrimental because it further increases the oxygen demand of the ischemic myocardium. In conditions that chronically elevate sympathetic activity, the cardiogenic sympathetic afferent reflex may potentiate the severity of an already life-threatening myocardial ischemia (Adamson and Vanoli, 2001). We have previously shown that the paraventricular hypothalamic nucleus (PVN) plays an important role in the cardiogenic sympathetic afferent reflex (Zahner and Pan, 2005; Zahner et al., 2024a) and that the PVN is activated by epicardial bradykinin (Xu et al., 2013). However, the role of central forebrain nuclei, particularly within the hypothalamus, are not fully known.

The dorsomedial hypothalamus (DMH) plays an important role in the sympathetic regulation of blood pressure and heart rate, thermogenic and metabolic activity, and serves as an integrating center for cardiovascular activity and physiological response to stress (Spyer, 1994; Fontes et al., 2001; DiMicco et al., 2002; Wang et al., 2006; Conceição et al., 2017; Ginty et al., 2017; Kono et al., 2020; Nakamura et al., 2022). The DMH sends projections to medullary nuclei involved in sympathetic control, such as the nucleus tractus solitarius, rostroventrolateral medulla (RVLM), and medullary raphe nucleus (Markgraf et al., 1991; Thompson and Swanson, 1998; Horiuchi et al., 2004; Womack and Barrett-Jolley, 2007). Activation of DMH neurons stimulates sympathetic activity that innervates the heart, blood vessels, and brown adipose tissue, which is mediated by differential projections of brain stem nuclei (Cao et al., 2004; Wang et al., 2010; Xavier et al., 2014). Disinhibition of the DMH by microinjection of bicuculline (a GABA_A_ receptor antagonist) increases basal blood pressure, heart rate, and renal sympathetic nerve activity (RSNA) similar to the response elicited by stress (DiMicco et al., 2002; Samuels et al., 2002; Cao et al., 2004) and augments the sympathetic baroreflex (McDowall et al., 2006).

There is good evidence to show that the DMH plays an important role in sympathetic cardiovascular regulation. Therefore, this study aimed to determine the role of the DMH in the cardiogenic sympathetic afferent reflex. To do this, we tested the reflex response to epicardial bradykinin in the anesthetized artificially ventilated naïve Sprague-Dawley rat before and after either activation or inhibition of the DMH. We tested the hypothesis that muscimol inhibition of the DMH attenuates and bicuculline disinhibition of the DMH augments the cardiogenic sympathoexcitatory reflex in the Sprague-Dawley rat. To show that the reflex response was mediated by DMH, we tested the response to epicardial bradykinin after bilateral microinjection of either the GABA_A_ agonist muscimol to inhibit or bicuculline to disinhibit DMH activity.

We found that while muscimol inhibition attenuated the peak arterial pressure and heart rate reflex response to epicardial bradykinin, there was no effect on RSNA, and the magnitude of the reflex response diminished. We also show that while bicuculline disinhibition of the DMH augmented the baroreflex-mediated increases in heart rate and RSNA, as previously described by McDowell et al., 2006, DMH disinhibition did not augment the response to epicardial bradykinin. These results demonstrate that while the basal activity of DMH may play a modest role in the full manifestation of the reflex response to epicardial bradykinin, increased sympathetic activity derived from the DMH does not augment the cardiogenic sympathetic afferent reflex in the naïve Sprague Dawley rat.

## 2 Methods

Adult male Sprague-Dawley rats (Envigo, Indianapolis, IN) 10–14 weeks old, weighing 300–400 g, were surgically prepared according to the Guide for the Care and Use of Laboratory Animals using procedures approved by the East Tennessee State University Committee on Animal Care and Use (protocol #P230601) (National Research Council, 2011). Data and analyses that support the findings of this study and additional details that support the findings of this study are available from the corresponding author upon reasonable request. Rats were initially anesthetized using 2% isoflurane in O_2_ through a nose cone to allow for the cannulation of vessels and tracheostomy. We confirmed the adequate depth of anesthesia before surgical preparations by the absence of withdrawal response to tail pinch. At the end of the experiments, rats were euthanized by an intravenous injection of an overdose of 1% αchloralose and decapitated.

### 2.1 Surgical preparation

We maintained each rat’s body temperature between 37°C and 38°C during the experiment with a heating pad and lamp coupled to a TCAT-2 temperature controller and rectal temperature probe (Physitemp Clifton, NJ). We shaved the ventral surface of the neck for a 4–5 cm incision along the midline, and the sternomastoid and sternohyoid were retracted laterally on both sides. We cannulated the left carotid artery to measure arterial pressure with a PT300 pressure transducer (Grass Instruments, Quincy, MA) connected to a P122 strain gauge amplifier (Grass Instruments, Quincy, MA). Heart rate was counted by triggering from the arterial pressure pulse. We cannulated the left jugular vein for the I.V. administration of anesthetics and paralytics (see below). The left and right femoral veins were cannulated for the separate administration of vasopressor drugs (for details, see “baroreflex procedure”). Next, we cannulated the trachea with a 14G endotracheal tube for mechanical ventilation using a rodent ventilator (CWE, Ardmore, PA). End-tidal O_2_ and CO_2_ were monitored with an O_2_ and CO_2_ gas analyzer (CWE, Ardmore, PA). Minute volume was set at 100 mL/kg, and CO_2_ was maintained at 4%–6% by adjusting the respiratory rate. A lateral thoracotomy was performed to expose the heart for the epicardial application of bradykinin.

A craniotomy was performed for the microinjection of drugs into the DMH. To do this, the rats were placed in a stereotaxic frame (Kopf Instruments, Tujunga, CA) with the incisor bar set to 5 mm below the interaural level. We removed the skin overlying the skull and exposed the surface of the brain by drilling a small square window (∼1 cm) corresponding to the coordinates of the DMH.

### 2.2 Renal sympathetic nerve recording

For renal sympathetic nerve recordings, we made a left flank incision. The left kidney was gently retracted laterally, and the left renal sympathetic nerve was carefully dissected from the surrounding tissue. The peritoneum was immersed in mineral oil, and the renal nerve was mounted on a bipolar hook electrode. The nerve signal was amplified (X10,000), and bandpass filtered (100–3,000 Hz) by an alternating current amplifier (model P511, Grass Instruments). The sympathetic activity signal was rectified, and lowpass filtered at a time constant of 0.5 s. The distal end of the renal nerve was cut to avoid recording any potential afferent activity.

After the laparotomy, rats were slowly administered 1% α chloralose (100 mg/kg I.V.) and weaned from isoflurane over approximately 20 min. This approach maintained a complete surgical anesthetic state and was verified by the absence of a corneal reflex or an increase in arterial pressure or RSNA to tail-pinch. After dissecting a renal sympathetic nerve and determining that the rats remained completely surgically anesthetized, they were paralyzed with D-tubocurarine (0.1 mg/kg I.V.) to block any spontaneous muscle twitching that typically occurs in the retracted external oblique. This dose of D-tubocurarine lasted ∼30–45 min and was allowed to wear off to assess the adequacy of the anesthetized state of the rat. In all cases, rats' arterial pressure, heart rate, and RSNA remained completely unresponsive to corneal stimulation or tail pinch. At the end of the recordings, background electrical noise was determined by cutting the proximal end of the renal nerve, thereby removing efferent activity. The remaining activity was subtracted from the integrated values of the RSNA.

### 2.3 Microinjections

For bilateral DMH microinjections (50 nL), a glass pipette (tip diameter 20–30 μm) was advanced to the DMH within the right hemisphere and then the left hemisphere. The stereotaxic coordinates for the DMH were 3.0 mm caudal from bregma, 0.5 mm lateral to the midline, and 8.5 mm ventral to the dura. Drugs were mechanically ejected over 1 s using a calibrated microinjection system (Nanoject III, Drumond Scientific, Broomall, PA), and the meniscus of the injectant was monitored using an operating microscope. After the microinjection into the right DMH, the glass pipette remained in place for ∼1 min to ensure adequate drug diffusion. This dose of muscimol lasts more than 90 minutes (Morin et al., 2001), and we have had success using this sequential rather than simultaneous microinjection approach. After we withdrew the glass pipette from the right hemisphere, it was placed in the respective stereotaxic coordinates for injection into the left hemisphere and left in place ∼1 min after the microinjection. For unilateral DMH microinjections (bicuculline) we only injected into the left DMH.

### 2.4 Epicardial bradykinin

After a high-quality nerve recording was obtained, a 15-min stabilization period was given. Then, baseline blood pressure, heart rate, and RSNA were recorded for ∼15 min. To stimulate cardiac nociceptive afferents, we applied bradykinin (10 μg/mL; Sigma, St. Louis, MO) to the anterior epicardial surface (∼1 cm^2^) of the left ventricle with a cotton-tipped applicator through the exposed window in the left chest, as previously demonstrated (Li and Pan, 2000; Li et al., 2001; Zahner et al., 2003; Zahner and Pan, 2005; Zahner et al., 2024a; Zahner et al., 2024b). After each bradykinin application, we washed the heart using ∼5 mL of room temperature normal saline, and the arterial pressure, heart rate, and RSNA returned to baseline levels over ∼10 min. The responses to epicardial bradykinin were examined at least twice, separated by ∼10–15 min, to ensure reproducibility.

### 2.5 Baroreflex procedure

To ensure that the sympathetic regulation of heart rate and RSNA remained responsive after bicuculline microinjections, as previously demonstrated by McDowell et al. (2006), we performed baroreflex tests after bicuculine microinjections. Baroreflex tests were accomplished using successive ramped infusions of the α-adrenergic agonist phenylephrine (PE; 125 μg/mL) and the vasodilator sodium nitroprusside (SNP; 50 μg/mL), via the left and right femoral vein catheters as previously described (Zahner et al., 2011; Zahner and Schramm, 2011; Castillo et al., 2012). We administered SNP first, beginning at a rate of 2.5 mL/h and increasing by 2.5 mL/h approximately every 10 s until A.P. was 60 mmHg below the baseline. We administered P.E. immediately after SNP, beginning at a rate of 2.5 mL/h and increasing by 2.5 mL/h every 10 s until ∼60 mmHg above baseline. These infusions produced an approximately linear increase in A.P. from 60 mmHg below baseline A.P. to 60 mmHg above baseline A.P. at a rate of ∼1.5 mmHg/s. We quantified RSNA during baseline recording prior to SNP delivery and during the phenylephrine-induced increase in A.P. after the SNP nadir.

### 2.6 Histology

The microinjection location within the DMH were examined and confirmed histologically in all rats. All microinjections contained 5% rhodamine-labeled fluorescent microspheres (0.04 μm, Molecular Probes, Eugene, OR). After the experiments, brains were removed rapidly, fixed in 4% paraformaldehyde solution, and stored at 4°C until cut. Frozen 40-μm coronal sections were cut on a freezing microtome and mounted on slides. We imaged sections with an Olympus BX43 fluorescence microscope and an Orca-Spark digital camera (Hamamatsu, Bridgewater, NJ). Brightfield and fluorescent images were merged to analyze the location of the glass injection needle tip and the distribution of the injectant. Microinjection locations were plotted on standardized sections from the Paxinos and Watson atlas (Paxinos and Watson, 2007). Rats with micropipette misplacement outside the DMH were excluded from the analysis.

### 2.7 Data analysis

Arterial pressure, heart rate, and RSNA were recorded with Cambridge Electronic Design Micro1401 hardware and Spike 2® software (Cambridge, UK). After a high-quality nerve recording was obtained, an ∼15 min stabilization period was given, followed by a 5 min control period to normalize baseline and reflex RSNA before and after microinjection treatments. All RSNA was normalized to that initial control period for each rat and expressed as a percentage change relative to that control period as previously described (Zahner and Schramm, 2011; Zahner and Beaumont, 2020; Zahner et al., 2024a; Zahner et al., 2024b). To assess the arterial pressure, heart rate, and RSNA responses to epicardial bradykinin, measurements were averaged during 30 s of the baseline period before epicardial bradykinin. The peak arterial pressure, heart rate, and RSNA responses were measured immediately after the bradykinin application.

We analyzed and plotted the data with GraphPad Prism software. Values are presented as means ± SEM. We performed the Shapiro-Wilk test for normality. A QQ plot of the residuals is included in the supplementary material showing that the normality assumptions were met. Two-way repeated measures were used to compare treatment and bradykinin response within the group. We performed Šídák’s post-tests to compare the difference between group means when F values were significant. A paired *t*-test was used to determine a significant difference in the magnitude of the reflex response. *p* < 0.05 was considered statistically significant.

### 2.8 Experimental design

To determine if inhibition of the DMH attenuates the cardiogenic sympathetic reflex, we first recorded control reflex responses to epicardial bradykinin. See supplementary material for an outline and schematic of the experimental design. Control responses were examined at least twice, separated by ∼10–15 min to ensure reproducibility. For microinjection studies, arterial pressure, heart rate, and RSNA response to epicardial bradykinin application were tested before (control) and after either bilateral microinjection (50 nL) of vehicle (saline) or the GABA_A_ receptor agonist muscimol (0.5 nmol) into the DMH. Because bilateral stimulation was not required to elicit a sympathetically mediated increase in arterial pressure, heart rate, and RSNA, the GABA_A_ receptor antagonist bicuculline (40 pmol) was injected into the left DMH. Microinjections were performed in separate groups of rats such that each rat only received one treatment. In the bicuculline-injected rats, we performed a baroreflex test as a positive control to show that sympathetic activity was indeed able to increase after bicuculline microinjection.

## 3 Results

**FIGURE 1:**
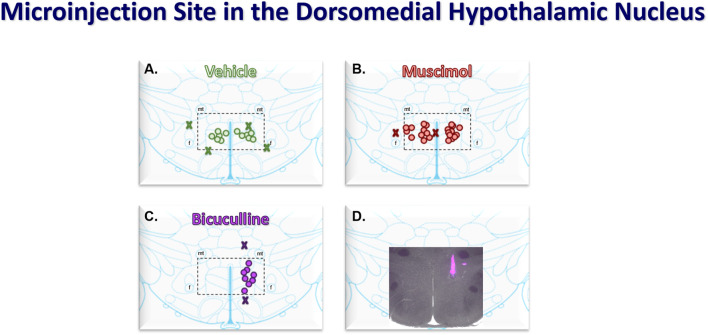
Coronal atlas images at the level of the DMH showing the compiled locations of bilateral (50 nL) microinjections of vehicle (saline, n = 6, **(A)** and muscimol (0.5 nmol, n = 11, **(B)** and the unilateral microinjections of bicuculline (40 pmol, n = 8, **(C)**. Injectants had 5% Fluorescent microspheres for histological verification. Merged fluorescent and light microscopic (2X) image showing a representative bicuculline microinjection **(D)**. Stereotaxic coordinates were 3 mm caudal to bregma, 0.5 mm lateral from the midline, and 8 mm ventral from the surface of the brain. The dashed rectangles medial fornix (f) and ventral to the mammillothalamic tract (mt) show the area of the dorsomedial hypothalamus (DMH).

We conducted these studies with a total of 31 anesthetized rats. We dismissed five rats from the grouped data due to inaccurate microinjection outside the DMH. A brief report of the physiological responses from those rats, as well as detailed statistical analysis and 95% confidence interval data, are included in the supplementary material. All microinjections were verified histologically and plotted according to Paxinos and Watson’s stereotaxic atlas (Paxinos and Watson, 2007) and located within 2.76–3.24 mm, caudal to bregma (Figures 1A–D). Consistent with previous reports, injections were considered within the DMH if the injection tip was medial to the fornix and ventral to the mammillothalamic tract (Poole et al., 2019). Supplementary Table 1 shows the grouped mean (±SEM) arterial pressure, heart rate, and RSNA values during baseline and the reflex response to epicardial bradykinin during control and after DMH microinjection treatment. Repeated bradykinin application consistently induced a response of similar magnitude as we have previously reported (Zahner et al., 2003; Zahner and Pan, 2005; Zahner et al., 2024a; Zahner et al., 2024b). Vehicle microinjection did not affect arterial pressure, heart rate, or RSNA during baseline or the reflex response to epicardial bradykinin (see Supplemental Results for details).

**FIGURE 2:**
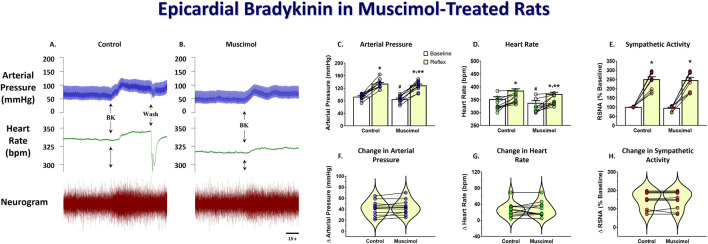
Original tracings showing arterial pressure, heart rate, and RSNA responses to epicardial bradykinin (BK, 10 μg/mL) during control **(A)** and after bilateral DMH microinjection (50 nL) of muscimol (0.5 nmol, **(B)**. Group data showing arterial pressure **(C)**, heart rate **(D)**, and RSNA **(E)** during baseline and the reflex response to epicardial bradykinin and the change in arterial pressure **(F)**, heart rate **(G)**, and RSNA **(H)** during control and after bilateral muscimol microinjection into the DMH. A repeated measures two-way ANOVA was used to identify a main effect of muscimol treatment or bradykinin application and interaction, followed by Šídák pos-hoc analysis. Paired *t*-test was used to identify differences in the change from baseline. * = significant increase from respective baseline, ** = significantly less than control response, # = significantly less than control baseline (n = 11, *p* < 0.05).

To determine the effect of DMH inhibition on the cardiogenic reflex, the response to epicardial bradykinin was tested before and after bilateral microinjection of the long-lasting GABA_A_ receptor agonist muscimol into the DMH. Figure 2 shows representative arterial pressure, heart rate, and RSNA during baseline and the reflex response to epicardial bradykinin during control (Figure 2A) and the baseline and the reflex response to epicardial bradykinin ∼10 min after bilateral microinjection of muscimol into the DMH (Figure 2B). Prior to muscimol microinjection, control epicardial bradykinin application significantly increased arterial pressure, heart rate, and RSNA (*p* < 0.001, Figures 2C–E). Bilateral muscimol microinjection into the DMH significantly reduced baseline arterial pressure and heart rate (*p* < 0.001). Muscimol microinjection into DMH did not significantly decrease baseline RSNA nor the bradykinin-induced increases in RSNA (*p* > 0.999, Figures 2E, H). While muscimol inhibition of DMH attenuated the magnitude of the bradykinin-elicited arterial pressure and heart rate response, the changes from baseline were not affected (*p* > 0.999, Figures 2F–H).

**FIGURE 3:**
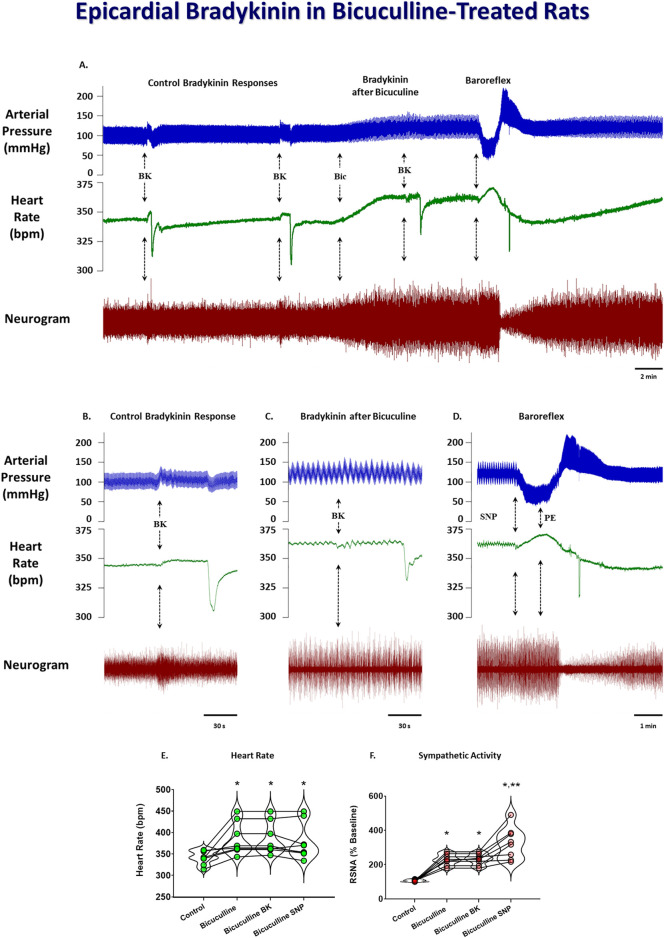
Original tracings showing arterial pressure, heart rate, and neurogram of renal sympathetic nerve activity responses to epicardial bradykinin (BK, 10 μg/mL) during control and after unilateral 50 nL microinjections of 40 pmol bicuculline **(A)**. The arrows indicate epicardial bradykinin (BK) application, bicuculine microinjection (Bic), and baroreflex testing with sodium nitroprusside (SNP) and phenylephrine (PE). Insets indicate reflex responses during control **(B)**, after bicuculline microinjection **(C)**, and baroreflex activity after bicuculline treatment **(D)**. Grouped data showing the heart rate **(E)** and RSNA **(F)** during control, after bicuculline microinjection, bradykinin application after bicuculline microinjection, and baroreflex unloading after bicuculline microinjection. A repeated measures one-way ANOVA was used to identify a significant effect, followed by Šídák pos-hoc analysis. * = significant increase compared with respective control, ** = significant increase compared with bicuculline microinjection (n = 8, *p* < 0.05).

**FIGURE 4:**
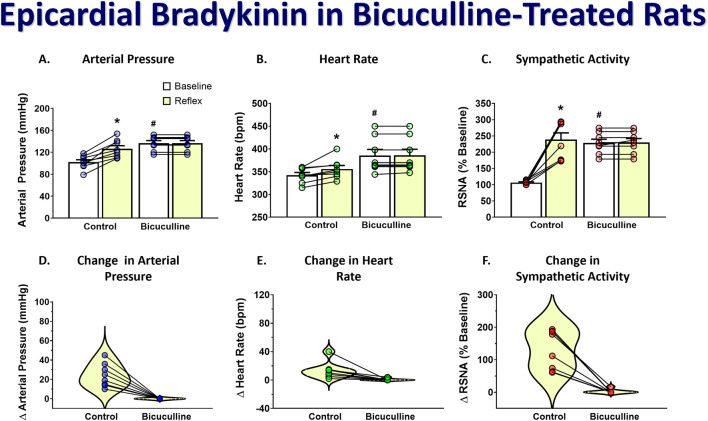
Group data showing arterial pressure **(A)**, heart rate **(B)**, and RSNA **(C)** during baseline and the reflex response to epicardial bradykinin and the change in arterial pressure **(D)**, heart rate **(E)**, and RSNA **(F)** during control and after unilateral microinjection (50 nL) of bicuculline (40 pmol). A repeated measures two-way ANOVA was used to identify a main effect of bicuculline treatment or bradykinin application and interaction, followed by Šídák pos-hoc analysis. Paired *t*-test was used to identify differences in the change from baseline. * = significant increase from respective baseline, * = significant increase from respective baseline, # = significantly greater than control baseline (n = 8, *p* < 0.05).

To determine if stimulation of the DMH augments the cardiogenic sympathetic reflex, we tested the reflex response to epicardial bradykinin application before and after bicuculline microinjection into the DMH. Because moderate bicuculline DMH disinhibition has been shown to augment the sympathetic baroreflex, and this was accomplished with a single unilateral DMH microinjection (McDowall et al., 2006), we only injected it into the left DMH for bicuculline microinjections. Bicuculline microinjections (40 pmol) into the DMH significantly increased baseline arterial pressure, heart rate, and RSNA. Figure 3 shows representative arterial pressure, heart rate, and RSNA during baseline and the reflex response to epicardial bradykinin during control (Figures 3A, B) and the baseline and the reflex response to epicardial bradykinin ∼2 min after microinjection of bicuculline into the DMH (Figures 3A, C). Prior to bicuculline microinjection, control epicardial bradykinin application significantly increased arterial pressure, heart rate, and RSNA (*p* < 0.001, Figures 4A–C). However, while bicuculline microinjection into the DMH significantly (*p* < 0.001) increased arterial pressure, heart rate, and RSNA from baseline, it did not augment the reflex responses to epicardial bradykinin (*p* > 0.999 Figures 4D–F). To show that increased sympathetic reflex activity is possible after muscimol disinhibition of neurons in the DMH, as previously demonstrated (McDowall et al., 2007), we performed baroreflex tests after DMH bicuculline. A representative tracing showing the baroreflex changes RSNA evoked by changes in arterial pressure induced by infusion of SNP and P.E. after microinjection of bicuculline methionine (40 pmol) into the DMH is shown in Figures 3A,D. While SNP-induced baroreceptor unloading after bicuculline treatment further increased RSNA it did not augment the bicuculline-induced tachycardia (Figures 3E, F).

## 4 Discussion

This study focused on the role of the DMH in the cardiogenic sympathetic reflex. Although the DMH has been well characterized in the sympathetic cardiovascular response to stress, its role in the sympathetic cardiovascular response to noxious stimulation of visceral sensory afferents is not known. While the arterial pressure and RSNA response to epicardial bradykinin application are mediated by presympathetic neurons within the rostroventrolateral medulla, the reflex tachycardia involves other supraspinal nuclei (Zahner et al., 2024b). In this study, we found that inhibition of the DMH using microinjection of the GABA_A_ receptor agonist muscimol caused a modest but significant decrease in basal arterial pressure and heart rate and attenuated the maximal reflex response to epicardial bradykinin. However, inhibition of DMH did not reduce basal RSNA nor diminish the reflex RSNA response to epicardial bradykinin.

While differential activation of sympathetic nerves has been shown to produce regionally specific sympathetic cardiovascular activity, we were surprised to observe that the inhibition of DMH did not affect RSNA. We considered the possibility that the muscimol microinjection could have spread to the paraventricular nucleus, which is one of the five presympathetic loci and shown to play an important role in generating and maintaining cardiovascular-related sympathetic activity (Strack et al., 1989). However, we ruled that out after careful mapping of the small volume of microinjection used (50 nL). Another consideration to rule out the possibility that muscimol microinjection decreased blood pressure and heart rate via spread into the PVN is that muscimol inhibition of PVN decreases heart rate and blood pressure by decreasing sympathetic activity. As such, we would have observed, as we have in the past, a decrease in RSNA and attenuated reflex response to epicardial bradykinin (Zahner and Pan, 2005; Zahner et al., 2024a). While it is tempting to speculate, it is more likely that the reduced cardiac output after DMH muscimol elicited baroreflex compensation of sympathetic activity. Whereas inhibition of PVN or RVLM directly inhibits cardiovascular-related sympathetic activity and directly reduces arterial pressure and heart rate, DMH influences sympathetic activity indirectly via projections to PVN, RVLM, and other brain stem nuclei. A similar decrease in basal heart rate evoked by bilateral muscimol injection into the DMH has been previously described (Lisa et al., 1989; Hunt et al., 2010).

It was previously shown that activation of DMH augments the sympathetic baroreflex (McDowall et al., 2006). In that study, they show that activation of the hypothalamic defense area via unilateral bicuculline microinjection increases basal sympathetic activity, blood pressure and heart rate and augments the baroreceptor-induced increases in RSNA. While the central components of the baroreflex are mainly mediated by nuclei within the brainstem medulla, projections from DMH either directly or indirectly modulate baroreflex-mediated sympathetic activity. Because stressful psychological stimuli (e.g., air jet stress) elicit a sympathoexcitatory cardiovascular response (DiMicco et al., 2002), it is likely that this augmented response is mediated at least in part by stimulation of barosensitive DMH neurons. In this study, we sought to determine if DMH stimulation would also augment the reflex response to nociceptive stimulation of cardiac afferents. To do this, we applied bradykinin to the epicardial surface of the heart to elicit a sympathetic reflex response during baseline control and after disinhibition of the DMH using unilateral microinjection of bicuculline. During the control recordings, epicardial bradykinin elicited a robust and repeatable increase in arterial pressure, heart rate, and RSNA. Bicuculline disinhibition of DMH increased baseline arterial pressure, heart rate, and RSNA, similar to that demonstrated by McDowell et al. (2006). However, while activation of DMH augmented baroreflex unloading-induced tachycardia and RSNA response, we showed that it did not affect the reflex response to epicardial bradykinin. While previous studies show that PVN plays an important role in the cardiogenic sympathetic reflex, data from this study shows that it is unlikely that DMH also serves an important role.

## 5 Perspective

While the sympathetic response to epicardial bradykinin is driven entirely by RVLM, we have previously shown that it is not wholly mediated by barosensitive RVLM activity (Zahner et al., 2024b). While it could be argued that disinhibition of DMH blocked the sympathetic response to epicardial bradykinin, an alternative explanation, albeit speculative, for why we did not see an augmented response to epicardial bradykinin after DMH stimulation is that bicuculline disinhibition stimulated the same population of neurons that are activated by epicardial bradykinin and that baroreceptor unloading elicited the increase in RSNA via separate population of neurons.

Whereas McDowall et al. (2006) show that both the baroreceptor unloading-induced increases in RSNA and heart rate are augmented after a single microinjection of 40 pmols bicuculline into the DMH, we only observed an augmented baroreceptor-induced increase in RSNA after the bicuculline microinjection into the DMH. Although the experimental conditions were very similar, one difference is that the studies by McDowall et al. (2006) were performed in rats anesthetized with urethane while we used α-chloralose. In this regard, it is possible that the anesthetic agent affected the ability to augment the reflex tachycardia. Although some show that baroreceptor reflex responses are enhanced by α-chloralose anesthesia (Brown and Hilton, 1956; Armstrong et al., 1961), others have shown diminished sensitivity and attenuated reflex tachycardia (Cox and Bagshaw, 1979; Ishikawa et al., 1984). While these studies were performed in dogs and rabbits, we are unaware of data comparing chloralose anesthesia with urethane in the rat. Notwithstanding, in our study, baroreceptor unloading after bicuculline microinjection into DMH increased RSNA, demonstrating that sympathetic activity could be increased. However, while we showed that modulation of DMH activity does not influence the magnitude of the cardiogenic sympathetic reflex, it should be considered that these experiments were performed in the acute rat model. As such, chronic stimulation or inhibition of DMH elicits central plasticity that may influence this sympathetic reflex response.

## Data Availability

The raw data supporting the conclusions of this article will be made available by the authors, without undue reservation.
