# Supplementary material for: The role of the dorsomedial hypothalamus in the cardiogenic sympathetic reflex in the Sprague Dawley rat
Source: Front Physiol. 2024 Dec 24;15:1479892. doi: 10.3389/fphys.2024.1479892 (PMC11703967; doi:10.3389/fphys.2024.1479892)
Supplement: Supplementary file 1 [file Table1.pdf]

Table. Cardiogenic Sympathetic Reflex After DMH Microinjection

	Blood Pressure (mmHg)				Heart Rate (bpm)				RSNA			
	Control		Treatment		Control		Treatment		Control		Treatment	
	Baseline	Reflex	Baseline	Reflex	Baseline	Reflex	Baseline	Reflex	Baseline	Reflex	Baseline	Reflex
Vehicle (n=6)	103.3 ± 5.6	132.0 ± 9.2 *	107.8 ± 5.1	137.0 ± 7.8 *	353.3 ± 7.5	370.6 ± 9.3 *	352.3 ± 7.8	367.4 ± 10.3 *	101.1% ± 0.9%	232.7% ± 15.2% *	102.3% ± 3.8%	226.6% ± 19.4% *
Muscimol (n=11)	93.0 ± 2.8	134.3 ± 4.1 *	85.2 ± 3.4 #	128.5 ± 4.5 *,**	332.0 ± 7.3	358.1 ± 5.6 *	320.3 ± 6.9 #	347.6 ± 5.6 *,**	100.1% ± 0.5%	250.7% ± 13.9% *	95.5% ± 3.5%	246.8% ± 14.9% *
Bicuculline (n=8)	102.0 ± 4.4	126.3 ± 5.8 *	136.6 ± 4.7 *	136.6 ± 4.7	342.4 ± 5.8	355.9 ± 7.6 *	385.5 ± 13.4 *	386.0 ± 13.2	106.4% ± 2.2%	238.6% ± 20.7% *	228.1% ± 11.5% *	230.2% ± 11.5%

* . = significantly greater than its respective baseline

** . = significantly less than its respective control reflex

\# . = significantly less than its respective control baseline
