# Supplementary figures and images for: The role of the dorsomedial hypothalamus in the cardiogenic sympathetic reflex in the Sprague Dawley rat
Source: Front Physiol. 2024 Dec 24;15:1479892. doi: 10.3389/fphys.2024.1479892 (PMC11703967; doi:10.3389/fphys.2024.1479892)

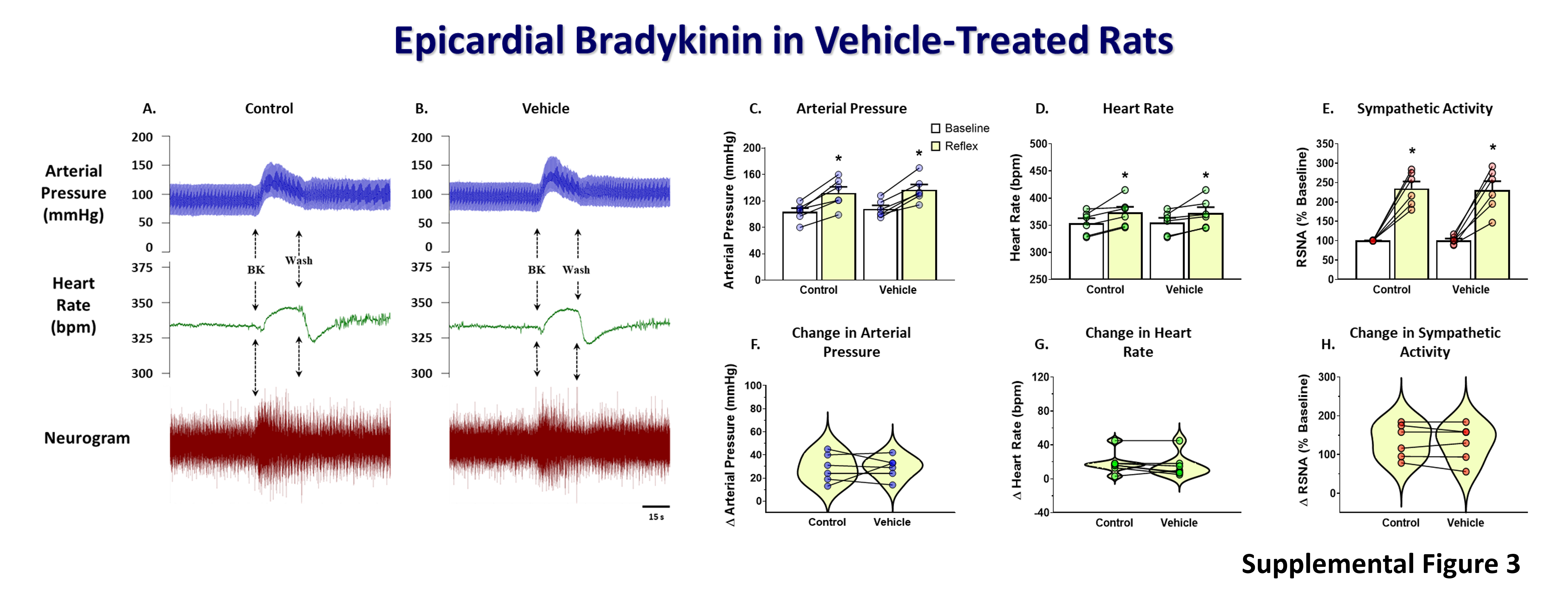

Supplement: Supplementary file 2 [file Image3.tif]

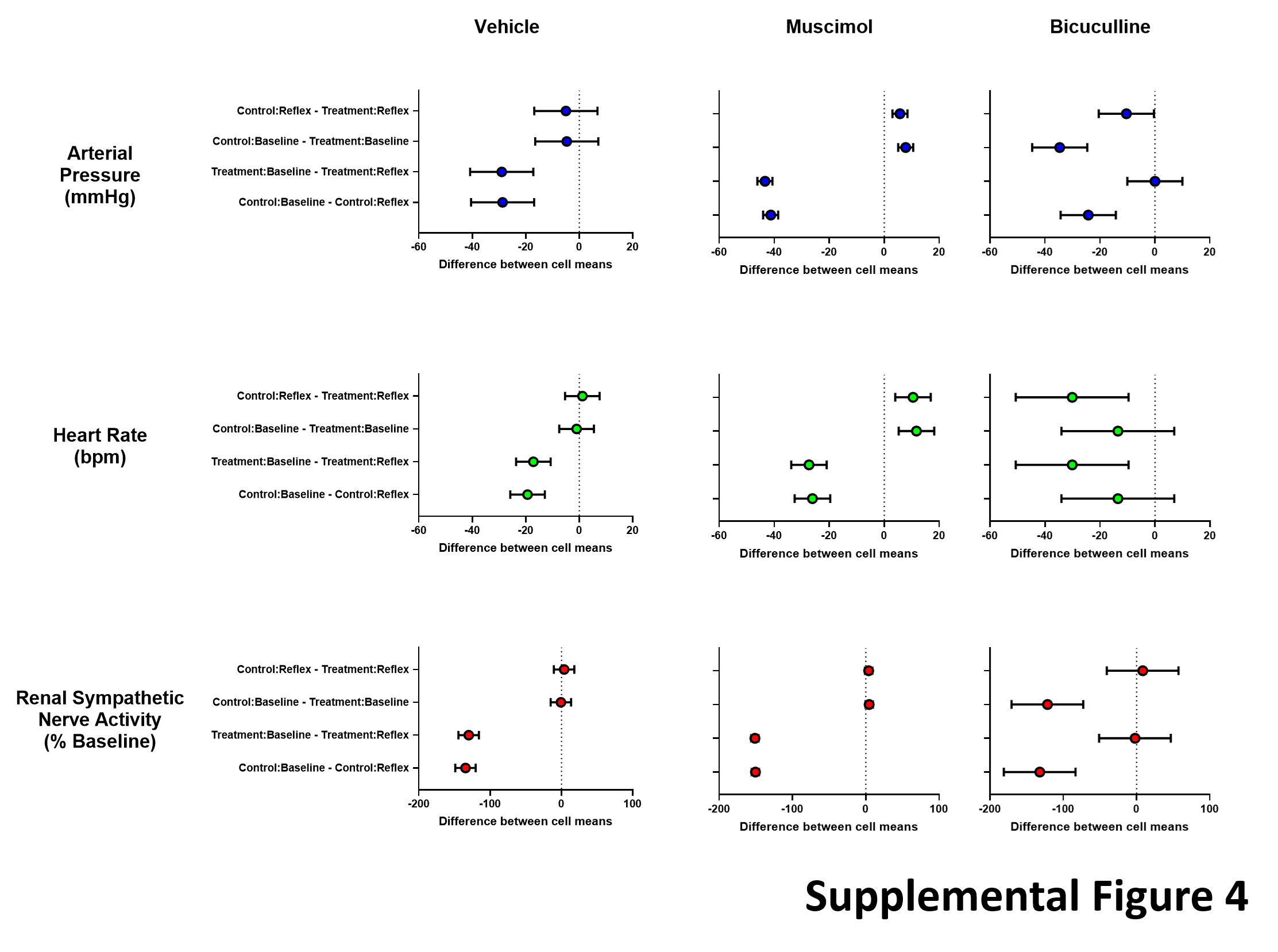

Supplement: Supplementary file 3 [file Image4.tif]

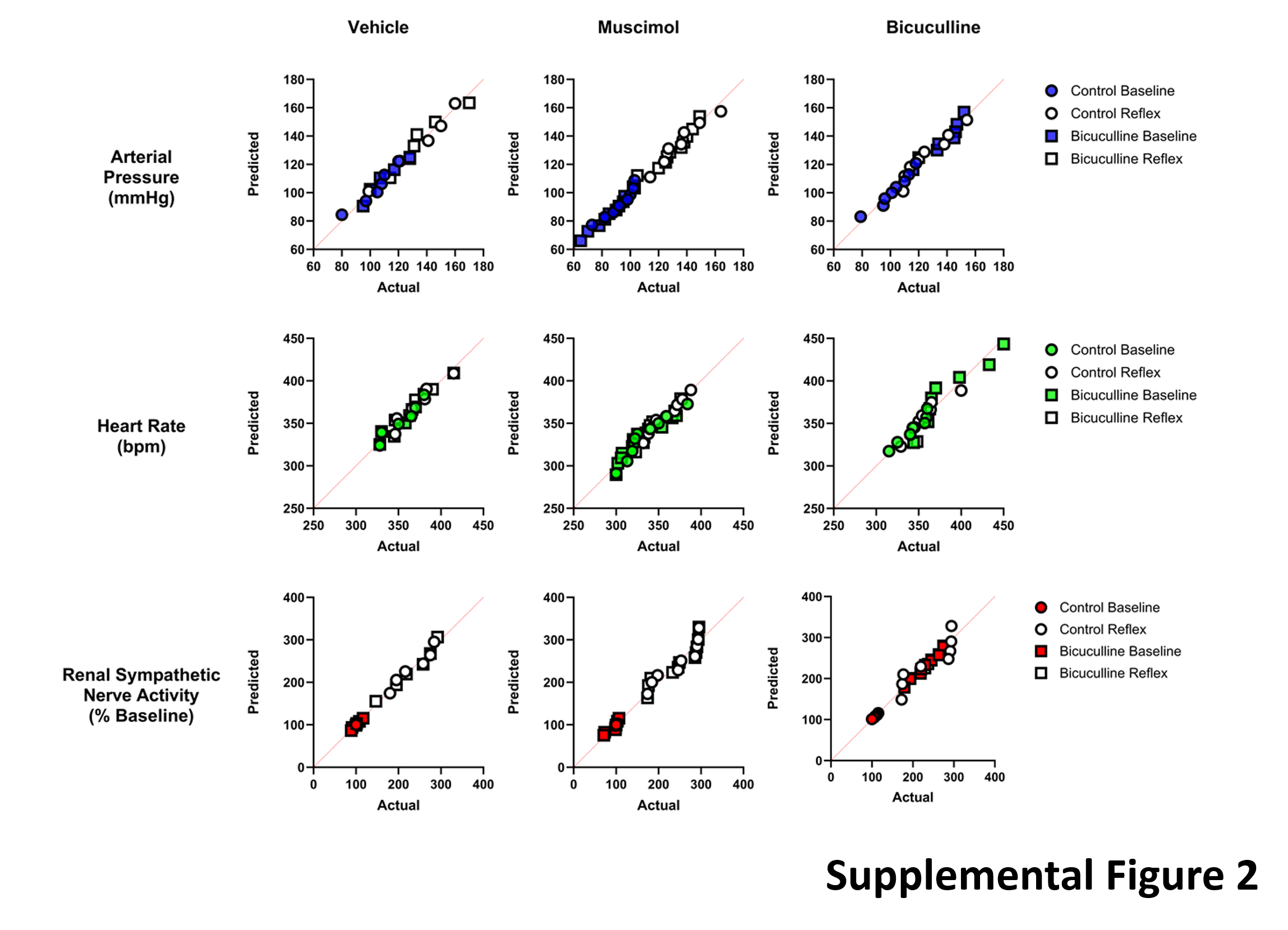

Supplement: Supplementary file 4 [file Image2.tif]
