# Supplementary material for: The role of the dorsomedial hypothalamus in the cardiogenic sympathetic reflex in the Sprague Dawley rat
Source: Front Physiol. 2024 Dec 24;15:1479892. doi: 10.3389/fphys.2024.1479892 (PMC11703967; doi:10.3389/fphys.2024.1479892)
Supplement: Supplementary file 5 [file Supplementaryfile1.pdf]

THE ROLE OF THE DORSOMEDIAL HYPOTHALAMUS IN THE CARDIOGENIC SYMPATHETIC REFLEX IN THE SPRAGUE DAWLEY RAT

Matthew R. Zahner[1*], Kynlee J. Hillard[1], & Michelle C. Chandley[2]

Supplemental Results

To ensure that DMH microinjection alone does not affect the cardiogenic sympathetic reflex, the response to epicardial bradykinin was tested before and after bilateral microinjection of vehicle (saline, 50 nl) into the DMH in a group of 6 rats. The supplemental figure 3 shows representative arterial pressure, heart rate, and RSNA during baseline, the reflex response to epicardial bradykinin during control and the baseline, and the reflex response to epicardial bradykinin before and ~10 minutes after bilateral microinjection of the vehicle into the DMH. Grouped data showing the 95% confidence intervals is shown in supplemental figure 4. Two-way repeated measures ANOVA of the grouped blood pressure, heart rate, and RSNA data during control and vehicle treatments revealed a main effect of epicardial bradykinin on blood pressure [$F(1, 5) = 54.1$, $P < 0.001$], heart rate [$F(1, 5) = 10.6$, $P = 0.022$], and RSNA [$F(1, 5) = 51.2$, $P < 0.001$]but no main effect of microinjection treatment on blood pressure [$F(1, 5) = 1.3$, $P = 0.298$], heart rate [$F(1, 5) = 0.003$, $P = 0.959$], or RSNA [$F(1, 5) = 0.1$, $P = 0.752$]. No significant treatment X bradykinin interaction was observed for blood pressure [$F(1, 5) = 0.006$, $P = 0.943$], heart rate [$F(1, 5) = 0.8$, $P = 0.411$], or RSNA [$F(1, 5) = 0.7$, $P = 0.432$]. Post hoc analysis indicates epicardial bradykinin application significantly increased arterial pressure, heart rate, and RSNA both before (control, $P< 0.001$) and after vehicle microinjection ($P < 0.001$, Supp Fig C-E). Paired t-test comparing the magnitude of the change in arterial pressure ($P = 0.916$), heart rate ($P = 0.222$), or RSNA ($P = 0.431$) before and after vehicle microinjection revealed no significant difference Supp Fig F-H).

Two-way repeated measures ANOVA of the grouped data revealed a main effect of muscimol treatment on blood pressure [$F(1, 10) = 5.1$, $P = 0.048$], heart rate [$F(1, 10) = 18.4$, $P = 0.002$], and RSNA [$F(1, 10) = 2.9$, $P = 0.121$], and a main effect of epicardial bradykinin on blood pressure [$F(1, 10) = 112.3$, $P < 0.001$], heart rate [$F(1, 10) = 25.9$, $P < 0.001$], and RSNA [$F(1, 10) = 112.5$, $P < 0.001$]. No significant treatment X bradykinin interaction was observed for blood pressure [$F(1, 10) = 2.7$, $P = 0.132$], heart rate [$F(1, 10) = 0.2$, $P = 0.682$], or RSNA [$F(1, 10) = 0.05$, $P = 0.821$]. Post hoc analysis indicates epicardial bradykinin application significantly increased arterial pressure, heart rate, and RSNA both before (control, $P< 0.001$) and after muscimol microinjection. Paired t-test comparing the magnitude of the change in arterial pressure ($P = 0.132$), heart rate ($P = 0.682$), or RSNA ($P = 0.822$) before and after vehicle microinjection revealed no significant difference.

Two-way repeated measures ANOVA of the grouped data revealed a main effect of bicuculline treatment on blood pressure [$F(1, 7) = 15.0$, $P = 0.006$], heart rate [$F(1, 7) = 11.3$, $P = 0.012$], and RSNA [$F(1, 7) = 27.4$, $P = 0.001$]. While there appeared to be a main effect of epicardial bradykinin on blood pressure [$F(1, 7) = 32.2$, $P < 0.001$], heart rate [$F(1, 7) = 11.3$, $P = 0.012$], and RSNA [$F(1, 7) = 36.8$, $P < 0.001$] this is likely due the interaction of bicuculline and bradykinin application observed for the arterial pressure [$F(1, 7) = 32.2$, $P < 0.001$], heart rate [$F(1, 7) = 9.8$, $P = 0.016$], or RSNA

[F(1, 7) = 39.0, P < 0.001]. Post hoc analysis indicates epicardial bradykinin application significantly increased arterial pressure, heart rate, and RSNA prior to bicuculline microinjection (control, P< 0.001) but not after bicuculline microinjection (arterial pressure and RSNA, P > 0.999; heart rate, P = 0.869; and RSNA, P = 0.889).

Vehicle microinjections missed the DMH in two rats (Fig 1A). In these two rats, vehicle injection, epicardial bradykinin increased mean arterial pressure from 109.5 ± 7.5 to 133.0 ± 17.0 mmHg, heart rate from 387.5 ±37.5 to 393.5 ±38.5, and RSNA from 109.6 ± 3.0 to 205.3 ± 14.4% of baseline. Microinjection of the vehicle that missed the DMH did not significantly alter bradykinin-induced increases in arterial pressure [F(3, 5) = 0.512, P = 0.691], heart rate [F(3, 5) = 0.84, P = 0.528], or RSNA [F(3, 5) = 1.97, P = 0.236]. After microinjection, epicardial bradykinin increased arterial pressure from 112.0 ±8.0 to 136.0 ±14.0 mmHg, heart rate from 382.5 ±45.5 to 386.5±46.5 bpm, and RSNA from 102.8 ± 7.2 to 185.2 ±14.8% of baseline. In the rat in which muscimol microinjections missed the right DMH, prior to microinjection, epicardial bradykinin increased mean arterial pressure from 90.7 to 132.8 mmHg, heart rate from 348.0 to 375.0, and RSNA from 104.3 to 239.1% of baseline. In that rat, muscimol microinjection into the left but not right DMH, epicardial bradykinin increased arterial pressure from 87.0 to 128.9 mmHg, heart rate from 348.0 to 375 bpm, and RSNA from 104.3 to 239.1% of baseline. Because the two bicuculline microinjections missed the DMH in two distinctly different nuclei, one microinjection was too deep and into the ventromedial hypothalamus, and the other was in the thalamic reuniens nucleus data from those rats were excluded from analysis.

Figure Legend

Supplemental Figure 1. Outline and schematic showing the experimental approach.

Supplemental Figure 2. Quantile-quantile plot showing the observed arterial pressure, heart rate, and renal sympathetic nerve activity (y-axis) during vehicle (n=6), muscimol (n=11), or bicuculline treatment (n=8) as a function of expected values from a normal distribution with the same mean and variance as the empirical distribution (x-axis).

Supplemental Figure 3. Original tracings showing arterial pressure, heart rate, and RSNA responses to epicardial bradykinin (BK, 10 µg/ml) during control (A), and after bilateral DMH microinjection (50 nl) of vehicle (saline, B). Group data showing arterial pressure (C), heart rate (D), and RSNA (E) during baseline and the reflex response to epicardial bradykinin and the change in arterial pressure (F), heart rate (G), and RSNA (H) during control and after bilateral vehicle microinjection into the DMH. A repeated measures two-way ANOVA was used to identify a main effect for vehicle treatment or bradykinin application and interaction followed by Šídák pos-hoc analysis. Paired t-test was used to identify differences in the change from baseline. * = significant increase from respective baseline (n=6, P < 0.05)

Supplemental Figure 4. Mean effect size (±95% confidence interval) of baseline and reflex response to epicardial bradykinin for arterial pressure, heart rate, and renal sympathetic nerve activity comparing after vehicle (n=6), muscimol (n=11), or bicuculline treatment (n=8).

This is a provisional file, not the final typeset article
