# Supplementary material for: The role of the dorsomedial hypothalamus in the cardiogenic sympathetic reflex in the Sprague Dawley rat
Source: Front Physiol. 2024 Dec 24;15:1479892. doi: 10.3389/fphys.2024.1479892 (PMC11703967; doi:10.3389/fphys.2024.1479892)
Supplement: Supplementary file 6 [file Image1.pdf]

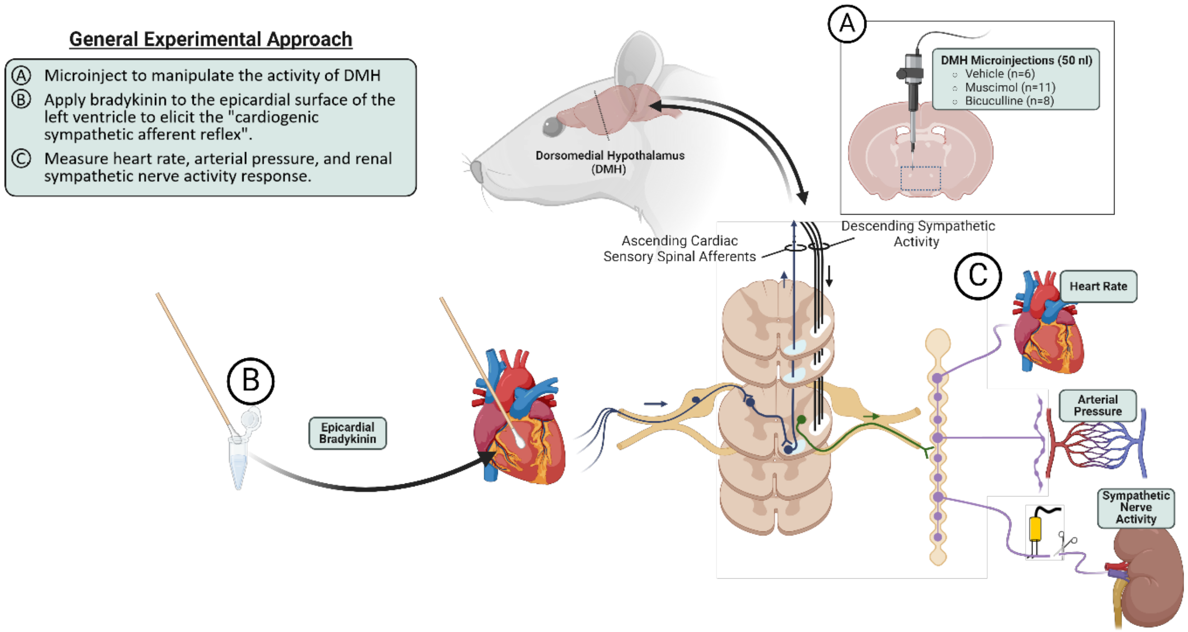

1) **Stabilization period from acute surgical preparation.**
2) **Control Bradykinin Tests**
   a) *Test "control CSAR" with epicardial bradykinin application.*
   b) *Wait for ~50-60 s, then wash bradykinin from the heart.*
   c) *Recovery of arterial pressure, heart rate, and RSNA to baseline (~10-15 min).*
   d) *Repeat "control CSAR" with epicardial bradykinin application.*
   e) *Wait for ~50-60 s, then wash bradykinin from the heart.*
   f) *Recovery of arterial pressure, heart rate, and RSNA to baseline (~10-15 min).*
3) **DMH Microinjections:**
   a) *Vehicle (saline, n=6)*
   b) *Muscimol (n=11)*
   c) *Bicuculline (n=8)*
4) **Control Bradykinin Tests**
   a) *Test "treatment CSAR" with epicardial bradykinin application.*
   b) *Wait for ~50-60 s, then wash bradykinin from the heart.*
   c) *Recovery of arterial pressure, heart rate, and RSNA to baseline (~10-15 min).*
   d) *Repeat "treatment CSAR" with epicardial bradykinin application.*
   e) *Wait for ~50-60 s, then wash bradykinin from the heart.*
   f) *Recovery of arterial pressure, heart rate, and RSNA to baseline (~10-15 min).*
5) **Perform baroreflex test in "bicuculline-treated" rats.**
6) **Euthanize and harvest the brain for histological verification of the injection site(s)**

Supplemental Figure 1
